# Influence of Benzyladenine on Metabolic Changes in Different Rose Tissues

**DOI:** 10.3390/plants7040095

**Published:** 2018-11-02

**Authors:** Mohammed Ibrahim, Xin Du, Manjree Agarwal, Giles Hardy, Muslim Abdulhussein, Yonglin Ren

**Affiliations:** 1 School of Veterinary and Life Science, Murdoch University, 90 South St., Murdoch, WA 6150, Australia; m.ibrahim@murdoch.edu.au (M.I.); B.du@murdoch.edu.au (X.D.); G.Hardy@murdoch.edu.au (G.H.); 2 Faculty of Agriculture, Al Qasim Green University, Babylon 51002, Iraq; 3 Faculty of Agriculture, University of Kufa, Najaf 54003, Iraq; muslim.alrubaye@uokufa.edu.iq

**Keywords:** rose, benzyladenine, headspace solid-phase microextraction (HS-SPME), VOCs

## Abstract

Two modern rose varieties, Floribunda and Hybrid Tea, were used to analyze and identify metabolic changes after foliar application with benzyladenine (BA). Volatile organic compounds (VOCs) as metabolites were detected. Two pairs of doses of BA, at 11.16 and 17.87 mg/cm^2^, and 7.17 and 12.26 mg/cm^2^ were applied to the foliage of Hybrid Tea and Floribunda, respectively. Sampling time was optimized and treatment duration was 4 weeks. After treatment, the volatiles from the treated and untreated control roses were extracted using headspace solid-phase microextraction (HS-SPME) technology by three-phase fiber 50/30 µm divinylbenzene/carboxen/polydimethylsiloxane (DVB/CAR/PDMS) and analyzed by gas chromatography (GC) coupled with a flame ionization detector (FID), and with mass spectrometry (GC-MS).The results showed that BA and its dose rate led to metabolic changes of treated roses in comparison with untreated controls. The number of VOCs extracted and detected from leaves, stem, rhizosphere and whole plants from the two rose varieties at doses rate of 17.87 and 12.26 mg/cm^2^ were 43, 65, 40 and 68 compounds for each plant material, respectively, for both rose varieties. Whilst the VOCs extracted and detected from both rose varieties for leaves, stem, rhizosphere and whole plants were 38, 61, 34 and 66 compounds for each plant material, respectively. The results demonstrate that some volatiles, such as 4-Heptyn-2-ol, Phenyl methyl ether and 3-Methyl-apopinene, increased with increasing doses of BA; these compounds are aroma chemicals with a very powerful smell. This study shows that BA treatments can have a significant effect on metabolite changes in different rose tissues. This method could be applied to other floriculture plants.

## 1. Introduction

The rose is one of the most common flowers cultivated in containers and gardens. It is important to the cut flower industry and for pharmaceutical purposes [1,2]. Thus, the rose is commercially very important. It is also highly sought after for interior decoration and for the perfume industry, which relies on different scented varieties [3,4,5,6,7]. There are numerous varieties of modern roses that are considered most important; among these varieties are Hybrid Tea and Floribunda [8]. 

Recently, plant growth regulators (PGRs) such as cytokinins (CKs) have become widely used to ensure efficient production of aromatic plants. There are many studies indicating that plant growth regulators affect the growth and development of various aromatic plants [9]. Ramesh et al. [10] reported that benzyladenine (BA), a cytokinin applied as a foliar spray at different concentrations, may reduce flower drop and senescence. Although PGRs have been used in agriculture and horticulture for decades, little is known about the effects of PGRs on the production of metabolic compounds [11]. 

Currently, volatile organic compounds (VOCs) have received special attention from the scientific community, as they have a wide range of biological and pharmacological uses and there are many publications related to the emission of volatiles [12]. Affonso et al. [13] treated *Lantana camara* plants with high concentrations (0.44 and 4.4 µmol/L) of BA and showed an increase in the production of volatile compounds detected by solid phase microextraction (SPME). These include myrcene, α-phellandrene, α-copaene, trans-caryophyllene and β-gurjunene. Another study indicated that incorporating plant growth regulators, such as 4.4 µM BA, 9.3 µM kinetin, 0.45 µM thidiazuron, 0.57 µM indole-3-acetic acid (IAA) and 0.41 µM picloram, into vitro media of *Agastache rugosa* plants increased volatile compounds, such as α-pinene, limonene, isomenthone and pulegone [14]. To date, no work has been conducted on the influence of BA on metabolite accumulation in roses. Moreover, there are few studies on modern rose varieties and the extraction of VOCs from different rose tissues by headspace solid phase microextraction (HS-SPME) [15]. HS-SPME analysis can be used to determine and monitor the composition of living natural materials or natural products and provide information on the olfactory profiles released from these [16]. The SPME technique has become increasingly common because it is a simple, fast and sensitive sample preparation method that reduces solvent usage while integrating sampling and sample preparation steps prior to instrumental analysis [17,18]. A recent study indicated that the HS-SPME technique can be successfully used to extract VOCs emitted from different rose tissues with the SPME fiber divinylbenzene/carboxen/polydimethylsiloxane (DVB/CAR/PDMS) coupled with gas chromatography (GC)–mass spectrometry (MS) [19]. However, there has been no work on the effect of BA on metabolic changes by using VOCs from rose plants. Therefore, the aim of this study was to increase the metabolite accumulation in two rose varieties through the addition of BA, and to determine this using HS-SPME with GC-flame ionization detector (FID)/MS. 

## 2. Materials and Methods

### 2.1. Standards and Reagents

Standard n-alkane (C7-C30) reference material, at 1000 μg/mL of each component in hexane, was purchased from Sigma-Aldrich (catalogue number 49451-U; Castle Hill, NSW, Australia). These included: decane, docosane, dodecane, eicosane, heneicosane, heptacosane, heptadecane, hexacosane, hexadecane, heptane, nonacosane, nonadecane, nonane, octacosane, octadecane, octane, pentacosane, pentadecane, tetracosane, tetradecane, triacontane, tricosane, tridecane and undecane. High-performance liquid chromatography grade ethanol was purchased from Merk (Kenilworth, NJ, USA). An n-hexane (95%) was purchased from Sigma-Aldrich (catalogue number 270504-2L; Castle Hill, NSW, Australia). Benzyladenine (BA) (98% purity, product number B3408) was purchased in crystalline form from Sigma-Aldrich (Castle Hill, NSW, Australia). Sodium hydroxide in pellet (97%) was purchased from Merck (catalogue number ME9M591064; Kenilworth, NJ, USA). Rooting hormone (Clonex) in gel form was purchased from Hydroponic Xpress (Code: 9313656614189; Perth, Western Australia, Australia). Vapor guard (anti-transpirant concentrate) liquid was purchased from Miller chemical (Hanover, PA, USA). Deionized water (Milli-Q Ultrapure water system) was obtained from Diversified Equipment Company (Lorton, VA, USA). Double distilled water was obtained from Refresh Pure Water (Perth, Western Australia, Australia). 

### 2.2. Apparatus and Equipment

Aromatic compounds of the 2 rose varieties were analyzed by an Agilent Technologies 7829A GC system (serial number CN14272038; Mulgrave, Victoria, Australia), fitted with a non-polar HP-5MS column (30 m × 0.25 mm, film thickness 0.25 μm, catalogue number 13423). The oven column temperature ranged from 50–250 °C, and it was programmed at 5 °C/min, with a final hold time of 5 min, using helium (He) as carrier gas at 1.1 mL/min constant flow, in a split ratio of 1:1. The GC was coupled with a flame ionization detector (FID). The GC-FID instrument was operated under the splitless mode, and the detector FID temperature was 290 °C. 

The volatile compounds were identified by GC-MS using an Agilent 7820A GC (Mulgrave, Victoria, Australia), equipped with a HP-5MS 30 m × 0.25 mm fused-silica capillary column (Santa Clara, CA, USA), with a film thickness of 0.25 μm, coupled to an Agilent model 5977E mass spectrometer detector (MSD) under splitless mode. The injector temperature was maintained at 250 °C. The GC conditions in the GC-MS analysis were the same as for the GC analysis, by using the pure chromatography (EZGC) method translator from the Restek website to translate the conditions of the GC-FID to GC-MS. The carrier gas He was supplied by BOC Gas (Sydney, Australia). The total GC-MS run time was 45 min and the temperature of the injector port was 250 °C. The VOCs were identified by matching their spectra with those recorded in a Mass Spectral library (National Institute of Standards and Technology (NIST) mass spectral search program for the NIST/EPA/NIH Mass Spectral Library version 2.2, 2014, Wiley, Hoboken, New Jersey, USA). 

In addition, the VOCs were confirmed by comparing the Kovats Indices or GC retention time data with those of authentic standards or from the published literature. A 50/30 µm divinylbenzene/carboxen/polydimethyl siloxane (DVB/CAR/PDMS, catalogue number 57347-U; Sigma-Aldrich Castle Hill, NSW, Australia)) fiber was attached to a manual SPME holder (Supelco Inc, Sigma-Aldrich Castle Hill, NSW, Australia). Three SPME fibers of medium polarity were used for extraction and the fibers were conditioned at the manufacture’s recommended conditioning temperature (270 °C) before analysis.

### 2.3. Plant Material and Maintenance

The experiment was carried out in March 2018 (summer). Ninety two-year-old rose plants grown in free-draining containers were used. These plants were prepared from cuttings of Hybrid Tea cv. Mr Lincoln and Floribunda cv. Iceberg, procured from Roworth Rose Nursery (Perth, Western Australia, Australia) and treated with rooting hormone (Clonex) in gel form and planted into seedling trays containing peat: perlite (1:1 *v*/*v*). The trays were then placed in a greenhouse at Murdoch University. After three months, the rooted cuttings were transferred to individual 16 cm × 16 cm (diameter × height) free-draining plastic pots. One year later, these were transferred to 24 cm × 24 cm (diameter × height) free-draining plastic pots consisting of two parts composted pinebark, two parts coarse river sand and one part coco peat, all obtained from Richgro (Perth, Western Australia, Australia). These were then placed in an evaporatively cooled glasshouse at Murdoch University. Glasshouse temperatures were maintained between 18 ± 2 °C and 25 ± 2 °C during the night and day, respectively, while the humidity was maintained at 60 ± 2% and 75 ± 2% day and night, respectively. 

The plants were fertilized every three months with a complete fertilizer (Miracle-Gro Maxfeed Soluble Plant Food All Purpose, including NPK) (Scotts Australia, NSW, Australia)) and watered manually daily to container capacity with about 400 mL of water. BA at 0 (control), 100, and 200 mg/L was applied to selected rose plants using a mist sprayer (450 mL). Benzyladenine powder (0 (control), 100 and 200 mg) was mixed with 4 to 6 drops of liquid sodium hydroxide (NaOH) 1 N and then added to 1 L of double distilled water. Each plant received 100 mL of solution through spraying, while the controls were sprayed with double distilled water. The containers were arranged in a factorial based completely randomized design with two rose varieties, two hormones, three concentrations of each hormone and three replicate plants for each treatment.

### 2.4. Determination of the Quantity of BA Sprayed on Plants by Using Filter Paper

To determine the actual dosage of BA applied to the plants, a filter paper (MACHEREY-NAGEL, Duren, Germany) was used. The filter paper was cut to different shapes and sizes according to leaves sourced from the top, middle and bottom of plants from both varieties of rose plants. The filter papers were weighed before used and then placed at the top, middle and bottom of the rose plants. After application of BA, as described above, the filter papers were removed and weighed again. This method was repeated twice and the average was taken (Table 1). Methods to quantify spray retention by leaves and the actual dosage, following the application of PGRs are still lacking, despite growing interest by researchers.

### 2.5. Sample Preparation and Extraction Using HS-SPME

At sampling, the rose plants were transferred to the laboratory. The samples were analyzed in biological triplicates. Three intact fully expanded and undamaged leaves, cut stems (13 cm long), rhizosphere soil and whole plants were sampled within different chambers for the extraction of VOCs as previously described [19]. Briefly, for leaf and stem samples, a 500 mL glass jar with septum, covered with aluminum foil was used. For rhizosphere sampling, a stainless steel probe 20 cm long with 1-millimeter diameter holes throughout and a septum installed in the top was inserted into the soil. SPME fiber was inserted through the septum. For whole plants, a glass chamber (30 × 35 × 60 cm) with a 5 mm port for the septum was used.

### 2.6. Optimization of Sample Collection Times after BA Application

**Figure 1:**
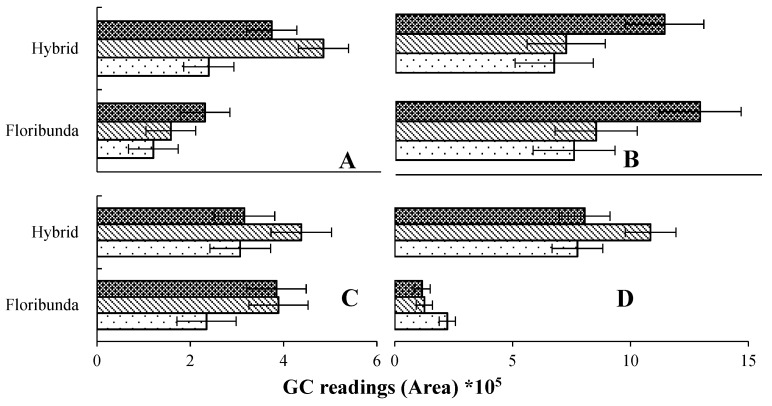
Comparison of main peak area of volatile compounds collected in different rose tissues; (**A**) leaves, (**B**) stems, (**C**) rhizosphere, (**D**) whole plant using different sampling times. 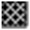 8 weeks, 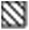 4 weeks and 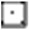 2 weeks, bars represent LSD at (*p* < 0.05) (*n* = 3).

A preliminary study was conducted to optimize the most efficient time for VOC sample collection after the application of BA to the rose plants, and three time periods (2, 4 and 8 weeks) were tested for each of the rose tissues (Figure 1). The samples from different rose tissues were prepared as described above.

### 2.7. Identification of Peaks and Compounds

Peaks were identified by using quantitative analysis software for GC identification. Total peak area for each treatment was compared with peak areas of the other treatments. Identified compounds from samples were also compared with the compounds identified from the internal standards. Identification of compounds was determined by comparing mass spectra and Kovatas Indices (KI) with authentic standards and data from published papers. GC-MS compounds, peaks, were identified using AMDIS version 2.72 (working under the NIST mass spectral search program for the NIST/EPA/NIH Mass Spectral Library) and by searching the NIST 2014 MS database with retention index confirmation. Relative percentage amounts of the separated compounds were calculated from total ion chromatograms by the computerized integrator.

### 2.8. Statistical Analyses

The results were analyzed statistically by SAS^®^ software, University edition, and the results were presented by analysis of variance (ANOVA). Means of 10 main peak areas (10 peak areas calculated together, divided by 10, to get the mean) were used to analyze and detect at different sampling times. Least significant difference (LSD) and the MetaboAnalyst 4.0 online program (http://www.metaboanalyst.ca/faces/home.xhtml) were used to compare means and significant differences reported at the 0.05 significance level.

## 3. Results and Discussion

### 3.1. Comparison of Optimal Sampling Times after BA Application

There were significant differences between the three sample times post BA application. Four weeks was selected as the optimal time for the absorption of volatile compounds from the leaves, rhizospheres and whole plants for both rose varieties (Figure 1A,C,D). In contrast, eight weeks was chosen as the optimum collection time of VOCs from stems (Figure 1B). These time periods were chosen as they gave superior total peak areas and numbers of compounds compared to the other sampling times. These findings for all tissues except stems agree with Baghele et al. [20] on *Rosa hybrida* cv Poison after application with BA. The collection time for stems corresponds with that found for *Aloe barbadensis* by Salehi Sardoei et al. [21], who found an optimum sampling time to be eight weeks after application with BA.

### 3.2. The Quantity of BA Applied to Rose Plants as Determined Using Filter Paper

The quantity of sprayed BA that lands on leaves can be affected by many factors. These include treatment efficiency, differences arising from retention variability depending on plant species, wettability, stage of growth, and architecture. Therefore, further investigation is needed on the effect of coverage and dosage on foliage as well as the methods to determine the actual dosage applied to the plant [22]. A few studies have used filter paper to detect and determine actual dosages of herbicides or insecticides applied to plants [23]. A study by Zabkiewicz et al. [24] reported that using filter paper showed that between 5% and 92% of an applied product can be off-target and not reach the leaves. There have been many field studies aimed at determining the best methods of herbicide and pesticide delivery based on volumes applied, actual doses, crop growth stage and species [25]. To our knowledge, there have been no studies on detection and quantification of actual dosage of PGRs after application to plant foliage.

The amount of droplets landing on the rose leaves was variable for practical reasons. When comparing the results for the whole plant, Hybrid Tea received a higher (11.16 mg/cm^2^) dose compared to Floribunda (7.17 mg/cm^2^) when treated with 200 mg/L BA (Table 1). The different locations of filter paper indicated the probability for different amounts of BA being retained. Leaves obtained from different parts of the rose canopy had different leaf area, with the top, middle and lower having the small, medium and large leaves, respectively. Thus, retention and adhesion of BA on the bottom leaves will be the greatest (Table 1). In addition, it may be because the distribution of filter paper, area spray volume and total retention of BA by the plant was different between the two roses. A study by Ramwell et al. [26] reported the use of filter paper for quantifying herbicides deposited or sprayed on plants. Actual dosage and retention of hormones depended on the amount of sprayed product, number of droplets landing on the leaf surface and leaf area [23]. It could be useful to relate how leaf angle might influence the overall retention in future studies.

### 3.3. Influence of BA on Rose Tissue VOCs

**Figure 2:**
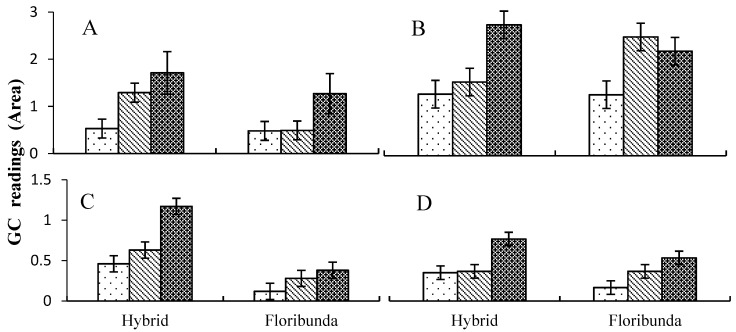
Effects of different applied concentrations of BA on the extraction efficacy of the total compounds identified from different rose tissues; (**A**) leaves, (**B**) stems, (**C**) whole plant and (**D**) rhizosphere. 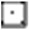 Control, 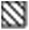 100 mg/L and 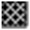 200 mg/L of BA; bars represent LSD at (*p* < 0.05) (*n* = 3).

The 200 mg/L BA application gave the highest peaks compared to 0 or 100 mg/L for the different rose tissues (Figure 2A,C,D). Similarly, a study by Abad Farooqi et al. [27] demonstrated that applying kinetin to *Rosa damascena* increased the amount of essential oils compared to control plants. Moreover, another study by Passinho-Soares et al. [28] indicated that BA applied to *Plectranthus ornatus* in plant tissue culture significantly increased VOCs and these helped callus induction. Prior to the current study, no work had been done on how BA affects VOCs production in vivo. 

### 3.4. Identification of VOCs Emitted from Different Rose Tissues

**Figure 3:**
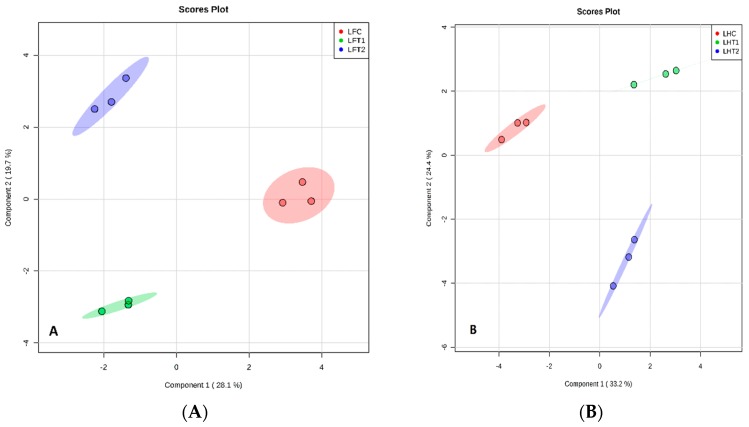
Sparse partial least squares discriminant analysis (sPLS-DA) model obtained from the classification of leaves from Floribunda and Hybrid Tea roses samples based on volatile organic compounds (VOCs) according to BA treatments. (**A**) Score plot of Floribunda and (**B**) score plot of Hybrid Tea leaves. Red, green and blue colors indicated groups of treatments. L represents Leaves, F represents Floribunda, H represents Hybrid Tea. While C represents control, T1 and T2 represent 100 and 200 mg/L BA, respectively. Three dots in each group mean *n* = 3 biological replicates.

**Figure 4:**
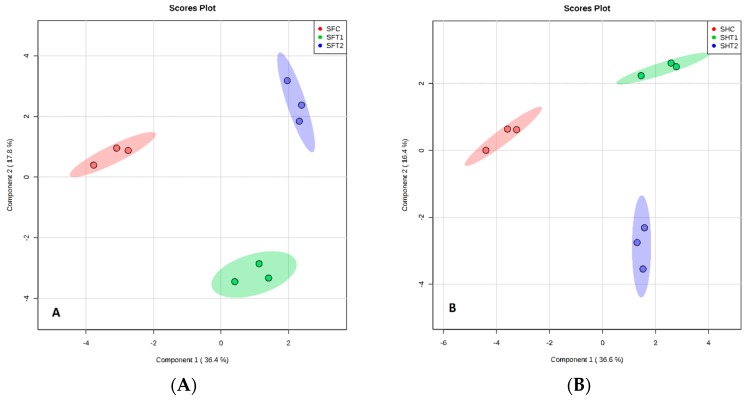
sPLS-DA model obtained from the classification of stems from Floribunda and Hybrid Tea roses samples based on VOCs according to BA treatments. (**A**) Score plot of Floribunda and (**B**) for the Hybrid Tea stems. Red, green and blue colors indicated groups of treatments. S represents Stems, F represents Floribunda, H represents Hybrid Tea. While C represents control, T1 and T2 represent 100 and 200 mg/L BA, respectively. Three dots in each group mean *n* = 3 biological replicates.

**Figure 5:**
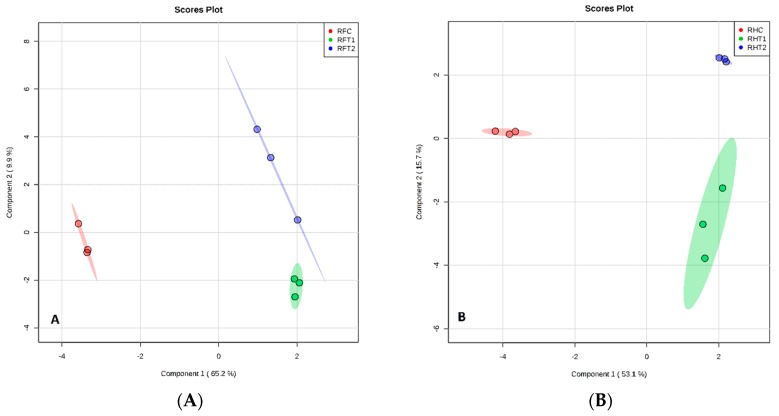
sPLS-DA model obtained from the classification of rhizosphere from Floribunda and Hybrid Tea roses samples based on VOCs according to BA treatments. (**A**) Score plot of Floribunda and (**B**) for the Hybrid Tea rhizosphere. Red, green and blue colors indicated groups of treatments. R represents rhizosphere, F represents Floribunda, H represents Hybrid Tea. While C represents control, T1 and T2 represent 100 and 200 mg/L BA, respectively. Three dots in each group mean *n* = 3 biological replicates.

**Figure 6:**
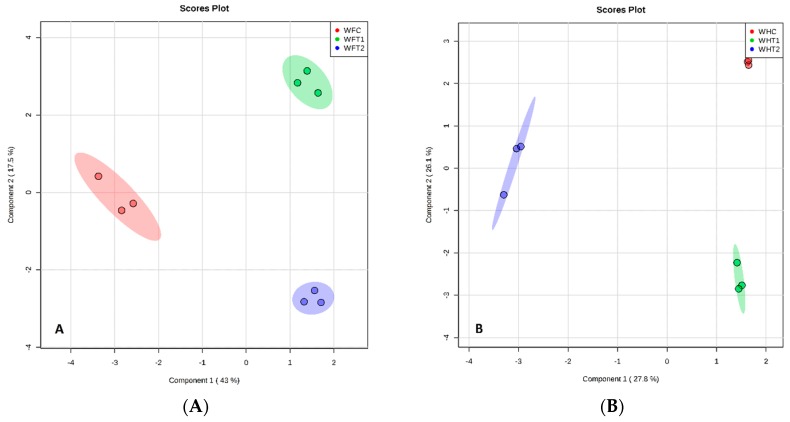
sPLS-DA model obtained from the classification of whole plants from Floribunda and Hybrid Tea roses samples based on VOCs according to BA treatments. (**A**) Score plot of Floribunda and (**B**) for the Hybrid Tea whole plants. Red, green and blue colors indicated groups of treatments. W represents whole plants, F represents Floribunda, H represents Hybrid Tea. While C represents control, T1 and T2 represent 100 and 200 mg/L BA, respectively. Three dots in each group mean *n* = 3 biological replicates.

All plants release VOCs from leaves, stems, flowers and rhizopheres. The rose plants produce a diverse range of secondary metabolites from all tissues, as determined by the sparse partial least squares discriminant analysis (sPLS-DA) of the data, as shown by the distinct clustering of samples according to BA treatments (Figure 3A,B). This indicates that different VOCs are produced in response to the different BA concentrations. There was no overlap in the VOCs produced between the three treatments of BA from the different rose tissues based on sPLS-DA (Figure 3, Figure 4, Figure 5 and Figure 6), respectively. There was a good separation between the data used for analysis of different BA treatments and these gave variances in the scoreplots for all rose tissues. The major differences that were found between Floribunda and Hybris Tea were Hexanal, 3-Hexen-1-ol, acetate, (E)- and Cedrol; these components have been found in Hybrid Tea and were not found in Floribunda. In contrast, α-Pinene, (D)- and Caryophyllene have been found in Floribunda, but were not found in Hybrid Tea with different rose tissues.

Identification of the compounds produced was based on a spectrometric data comparison. A total of 43, 65, 40 and 68 VOCs were detected from leaves, stem, rhizospheres and whole plants, respectively. Only those VOCs shown to differ significantly between the three BA concentrations tested according to ANOVA with a p value of 0.05 were identified to a specific chemical compound. For Hybrid Tea, there were 4, 6, 1 and 5 compounds from leaves, stems, rhizospheres and whole plants, respectively; from Floribunda, there were 2, 6, 2 and 6 compounds, respectively (Table 2). 

Some VOCs increased as the BA levels applied increased, for example, 4-heptyn-2-ol, one of the precursors of fragrance compound (US 7,842,660 B2) was found to increase from 20.26 to 54.43 in the total peak area as BA levels increased from 0 to 200 mg/L in whole plant tissue for both rose varieties (Table 2). In the case of Floribunda root tissue, phenyl methyl ether, an aromatic chemical used to produce essential oils, increased with increased BA. For leaves, the amount of 3-methyl-apopinene increased with increasing levels of BA in Hybrid Tea; this compound is an important essential oil and corresponds to the finding by Mahdavi et al. [29] on the extraction of essential oil from Etlingera brevilabrum leaves. In a study by Sun et al. [30] different chrysanthemum cultivars were shown to produce different types and numbers of volatiles compounds. A similar observation was made by Sparinska et al. [31] where different rose cultivars gave different types and numbers of VOCs. Furthermore, a study by Ahmad et al. [32] demonstrated that the SPME technique obtained more VOCs from *Polygonum minus* compare to the hydrodistillation technique. This difference may be due to the heat applied during hydro- distillation, which can degrade some heat unstable compounds; consequently, the difference can be attributed to the high sensitivity of the SPME technique. For eucalyptus leaves, SPME was successfully used to collect and identify VOCs [33]. Based on our results and those of previous studies, HS-SPME can be used to analyze volatile compounds emitted from fresh tissues [19].

## 4. Conclusions

Our study demonstrates that the collection of VOCs from fresh leaves, stems, rhizospheres and whole plants by HS-SPME coupled with GC-FID/MS represents a sensitive, rapid and effective method for analyzing and detecting VOCs. In total, 32 compounds were identified and the concentrations of these differed significantly depending on the different concentrations of BA applied to the roses. The highest concentration of VOCs for both rose varieties occurred four weeks after BA application, with significantly lower concentrations at two and eight weeks. Increased leaf area led to increased retention of BA, and consequently, an increase in the actual dosage of BA applied to the rose plants as determined by the filter paper method. In addition, different responses were observed for the four tissues of both rose varieties, as shown by the different VOCs detected after BA application. However, there were differences in the amounts of different volatiles between the rose tissues. Of interest to the industry, BA was shown to have a significant effect on rose metabolites, and some aromatically important compounds increased following the application of 100 mg/L or 200 mg/L BA. In addition, these compounds, 4-heptyn-2-ol, phenyl methyl ether and 3-methyl-apopinene, are precursors for making fragrant compounds. Consequently, BA can be used to increase and improve the quantity and quality of VOCs produced from roses and other floricultural plants. Thus, in the future, BA can be used to increase the fragrance of the flowers. 

## Figures and Tables

**Table 1:** The actual dosage (mg/cm^2^) of two benzyladenine (BA) formulations (100 & 200 mg/L) after application to two rose varieties as determined by using filter paper.

	Hybrid Tea (mg/cm^2^)		Floribunda (mg/cm^2^)	
Canopy Leaves	100 mg/L	200 mg/L	100 mg/L	200 mg/L
Top	8.82 ^b^	14.53 ^b^	7.70 ^a^	13.38 ^a^
Middle	12.43 ^a^	18.11 ^a^	5.76 ^a^	8.99 ^b^
Bottom	12.25 ^a^	20.98 ^a^	8.06 ^a^	14.42 ^a^
Whole plant	11.16 ^b^	17.87 ^a^	7.17 ^b^	12.26 ^a^
LSD	3.73	3.23	4.58	3.33

Similar letters mean there are no significant differences between different leaf level with least significant difference (LSD) *p* ≤ 0.05. Numbers are according to the equation: mg/cm^2^ = $\frac{Wa-Wb(\mathrm{mg})}{\mathrm{LA}({\mathrm{cm}}^{2})}$. (*Wa*) means weight after application, (*Wb*) means weight before application, while LA means leaves area

**Table 2:** Identification of compounds that differed significantly between different rose tissues after being treated with different concentrations of BA.

				Leaves			Stems			Rhizosphere			Whole plants		
				Mean of Peaks			Mean of Peaks			Mean of Peaks			Mean of Peaks		
RT	Compounds	RI	C	0	100	200	0	100	200	0	100	200	0	100	200
6.56	Hexanal	769	H	1.27	2.57	5.62	23.02	32.89	44.74 *	26.53	28.79	28.46	2.51	7.07	6.63
8.15	*cis*-β-Hexenyl formate	802	H	1.35	4.73	2.10	0.94	5.70 *	2.32	ND	ND	ND	3.66	3.57	3.98
9.42	Phenyl methyl ether ^†^	878	F	3.88	4.95	14.20	5.55	5.86	8.24	15.46	19.46	30.61 *	14.75	16.35	43.55
9.56	4-Heptyn-2-ol ^†^	897	H	5.73	7.63	13.22	ND	ND	ND	27.68	43.50	27.28	20.26	16.23	54.43 *
			F	ND	ND	ND	3.74	4.71	4.83	29.98	40.95	74.59	5.74	7.77	37.03 *
10.36	3-Methyl-apopinene ^†^	922	H	6.09	3.05	12.72 *	4.37	14.39	13.57	ND	ND	ND	2.28	2.85	4.56
10.44	α-Pinene, (D)-	931	F	4.25	12.64	30.66	4.73	4.53	5.51	0.17	0.69	0.25	1.95	6.43 *	2.01
12.72	3-Hexen-1-ol, acetate, (E)-	983	H	2.50	3.54	5.96	2.58	3.66	2.59	5.91	16.78	6.53	15.89	17.12	24.02 *
14.05	β-*cis*-ocimene	1005	F	2.31	2.92	2.70	1.42	2.09	5.97 *	ND	ND	ND	0.45	0.76	0.74
14.15	3-Carene	1024	F	ND	ND	ND	11.40	14.69	19.94 *	ND	ND	ND	0.34	0.43	0.56
15.96	Nonanal	1081	H	0.12	0.26	0.43	2.20	44.23 *	5.02	ND	ND	ND	0.57	0.56	0.59
17.02	α-Pyronene	1115	F	ND	ND	ND	4.81	5.14	6.75	ND	ND	ND	0.51	2.85	0.59
18.82	2,2-Dimethylpentane	1221	H	0.46	1.19	0.51	0.37	1.60	0.20	ND	ND	ND	0.23	1.24	1.66
20.27	Toluene, 3,5-dimethoxy	1245	H	1.72	2.02	1.83	ND	ND	ND	2.55	3.60	2.53	0.36	2.30	0.67
22.66	2,3-Dimethylundecane	1284	F	ND	ND	ND	2.33	3.77	3.18	ND	ND	ND	1.83	2.40	11.47 *
23.89	Copaene	1392	F	0.17	0.43	3.13 *	ND	ND	ND	1.32	5.00	1.43	0.18	0.18	2.73
24.40	β-Bourbonene	1408	H	0.60	7.02 *	0.34	ND	ND	ND	ND	ND	ND	0.33	0.65	1.31
24.76	2,4-Diisopropenyl-1-methyl-1-vinylcyclohexane	1416	H	0.33	0.38	0.46	0.20	0.40	0.56	0.72	1.37	0.86	4.31	9.11	13.15
			F	0.21	0.36	0.55	0.13	0.44	3.85	0.62	1.46	4.71	14.82	1.47	0.74
24.92	α-Cedrene	1423	H	0.20	0.20	1.05	2.86	3.34	2.89	0.32	0.41	0.31	0.17	4.11	0.71
			F	0.10	0.37	0.50	2.33	3.77	3.14	0.35	0.36	0.42	0.40	1.60	1.54
25.07	Caryophyllene	1431	F	0.21	0.36	0.55	0.29	0.38	0.47	0.52	0.56	2.64 *	0.35	0.59	0.36
26.10	beta-Guaiene	1469	F	0.27	0.47	2.67	0.38	2.53	5.07 *	ND	ND	ND	0.28	2.37	1.16
26.46	γ-Muurolene	1494	H	0.55	0.32	0.56	ND	ND	ND	ND	ND	ND	1.37	1.54	4.25 *
			F	ND	ND	ND	0.39	0.48	0.77	ND	ND	ND	2.36	3.16	6.71 *
27.14	Froggatt ether	1509	H	2.39	3.08	3.63	1.99	1.58	2.14	2.05	2.27	1.94	0.69	2.72	1.63
			F	1.82	3.38	1.23	2.14	2.25	3.76	ND	ND	ND	0.51	1.91	6.84
28.29	β-Calacorene	1548	F	ND	ND	ND	5.14	9.80	9.34	0.50	0.57	0.54	1.04	1.13	1.27
29.66	Cedrol	1595	H	1.58	3.24	1.53	0.26	0.60	0.49	0.53	1.38	4.62	0.46	0.60	0.72
34.48	N-Acetylhystrine	1935	H	1.15	1.29	1.39	0.45	0.48	0.49	ND	ND	ND	ND	ND	ND
			F	0.61	0.89	0.75	0.35	0.49	0.48	1.06	2.01	1.52	0.29	1.35	3.05

(*) Means: there are significant differences between different concentrations of BA (0(control), 100, 200 mg/L) with LSD *p* ≤ 0.05; (RT) retention time, (RI) retention index based on alkane series, (C) means cultivars of Roses (F) Floribunda and (H) Hybrid Tea, (ND) not detected. (†) Means: components that increased significantly in leaves, stems, rhizosphere and whole plants with increasing level of BA as compare to control.
